# Behavioral Activation for Prevention of Post-Stroke Depression in Low-Income Older Stroke Survivors (LIVE-WEL)

**DOI:** 10.1093/geroni/igaf122.1992

**Published:** 2025-12-31

**Authors:** Jennifer Beauchamp

**Affiliations:** UTHealth Houston, Houston, Texas, United States

## Abstract

Post-stroke depression (PSD) affects approximately one-third of survivors and is associated with recurrent stroke, poor recovery and quality of life, and mortality. The efficacy of antidepressants to treat PSD is based on mixed results and can be problematic for older survivors who can be sensitive to adverse effects and drug-drug interactions. Behavioral activation (BA) has been shown to be efficacious in treating depression in a range of populations and can be adapted for stroke survivors. This randomized effectiveness study was designed to examine the effectiveness of tele-delivered BA delivered by community health workers to prevent PSD in low-income (single person income ≤ 45,000), older (≥ 55 years) adults with subthreshold depression (SD; 24-item Hamilton Rating Scale for Depression [HAMD] between 5 to 8) within 3 months of a first-time ischemic or hemorrhagic stroke. Eligible survivors will be randomized (1:1) to the tele-delivered BA or treatment-as-usual. Tele-delivered BA will consist of 5-weekly videoconferences, homework, and 2-monthly booster calls. Treatment-as-usual includes an educational packet and a stroke practitioner evaluation within 6 weeks of hospital discharge that includes PSD screening. Antidepressant prescribing and/or referral to mental health care may be done according to practitioner practices as well as risk factor and complication assessments, stroke education, and care management. The primary outcome is the difference in the proportion of survivors that transition from SD to PSD at 4-months, assessed using generalized linear modeling. Secondary outcomes include anxiety, emotional distress, healthcare visits, quality of life, and disability. Mediating variables include self-efficacy, motivation, and activity engagement.

